# Many Is Better Than One: An Integration of Multiple Simple Strategies for Accurate Lung Segmentation in CT Images

**DOI:** 10.1155/2016/1480423

**Published:** 2016-08-22

**Authors:** Zhenghao Shi, Jiejue Ma, Minghua Zhao, Yonghong Liu, Yaning Feng, Ming Zhang, Lifeng He, Kenji Suzuki

**Affiliations:** ^1^School of Computer Science and Engineering, Xi'an University of Technology, Xi'an 710048, China; ^2^Xianyang Hospital, Yan'an University, Xianyang 712000, China; ^3^First Affiliated Hospital of School of Medicine, Xian Jiaotong University, Xian 710061, China; ^4^School of Information Science and Technology, Aichi Prefectural University, Nagakute, Aichi 480-1198, Japan; ^5^Medical Imaging Research Center, Illinois Institute of Technology, Chicago, IL 60616-3793, USA

## Abstract

Accurate lung segmentation is an essential step in developing a computer-aided lung disease diagnosis system. However, because of the high variability of computerized tomography (CT) images, it remains a difficult task to accurately segment lung tissue in CT slices using a simple strategy. Motived by the aforementioned, a novel CT lung segmentation method based on the integration of multiple strategies was proposed in this paper. Firstly, in order to avoid noise, the input CT slice was smoothed using the guided filter. Then, the smoothed slice was transformed into a binary image using an optimized threshold. Next, a region growing strategy was employed to extract thorax regions. Then, lung regions were segmented from the thorax regions using a seed-based random walk algorithm. The segmented lung contour was then smoothed and corrected with a curvature-based correction method on each axis slice. Finally, with the lung masks, the lung region was automatically segmented from a CT slice. The proposed method was validated on a CT database consisting of 23 scans, including a number of 883 2D slices (the number of slices per scan is 38 slices), by comparing it to the commonly used lung segmentation method. Experimental results show that the proposed method accurately segmented lung regions in CT slices.

## 1. Introduction

Accurate lung segmentation is very important to ensure the performance of computer-aided lung diseases diagnosis (CAD) systems [1]. A recent study shows that 17% of true positives were missed because of poor lung segmentation [2]. Hence, there has been a growing interest in automated and accurate segmentation methods for lung CT images in recent years. Studies have reported on many methods, which are generally classified as threshold-based [3], region-based [4–6], and deformable-model-based methods [7–10]. Though they provide good results, no method has demonstrated robust and accurate results across the wide range of clinical imaging parameters and pathology faced in clinical practice. And so it remains an ongoing challenge to segment lung regions accurately in CT slices because of the complex anatomy of the thorax and image artifacts.

In this paper, a novel method for accurately segmenting lung in CT (computed tomography) slice based on the integration of multiple segmentation strategies was proposed. The method was derived of several different simple strategies. Firstly, in order to avoid noise disturbances, the input CT slice was smoothed using a guided filter. Then, the smoothed slice was transformed into a binary image using an optimized threshold. Next, a region growing and random walk strategy was employed to obtain the masks of the lungs. Finally, with the lung masks, lung regions were automatically segmented from a CT slice. The proposed method was assessed on 23 lung CT scans with 883 2D slices. Experiments indicate that this method achieved an average absolute border distance of 0.62 mm compared to manually segmented ground truths.

The primary contributions of the paper are summarized as follows:

1. A fully automatic approach for accurate lung segmentation is developed by effective integrating multiple well developed simple image preprocessing strategies. The whole process does not need any user interaction.
2. The affection of image noise on integrity of lung segmentation is counted.
3. To make the segmentation of lung region more accurate, the contours of initial segmented lungs are refined using a curvature-based correction method. The proposed method can include all juxtapleural nodules near the mediastinum.

The remainder of this paper is organized as follows. To easy understand the value of this work, related works are discussed in Section 2. The proposed method is described in Section 3. Subsequently, experimental results are presented and discussed in Sections 4 and 5, respectively. Finally, conclusions and further discussion are provided in Section 6.

## 2. Related Works

One of the most commonly used lung segmentation methods for CT images is the threshold-based method [3], where the contrast between the low-density lungs and the surrounding high-density chest wall is usually used to guide the segmentation process, and by which tissues having higher gray levels than the selected threshold are excluded from the thoracic region. Although threshold-based strategies can be used to extract the lung parenchyma, these methods may fail in intensity variation condition and incorrectly exclude some vital regions, for example, juxtapleural nodules, from the lung area. Region-based methods, such as region growing [4] and graph cutting [5], are useful for dealing with intensity variations. However, with the presence of dense pathology in the lung field, it is not enough for successful delineation only with intensity. Deformable-model-based methods are widely used for the segmentation of pathological lungs [9, 10]. For example, in [9], an initial contour close to the lung boundary is firstly obtained, and then the contour reaches the object border. The limitation of this method is that some edge information might be lost when the edge of the contour is cracked. In [10], the level set method is employed to find the lung boundaries using energy minimization procedures. Though these methods are accurate in segmenting lung regions, they might lead to local minima states.

Recently, random-walk-based segmentations [12], in which graph optimization is employed to obtain accurate segmentation with user interaction based on selected seed pixels, are attracting increasing interest. Though this method shows excellent performance in image segmentation, it is sensitive to seed pixels.

## 3. The Proposed Method

**Figure 1:**
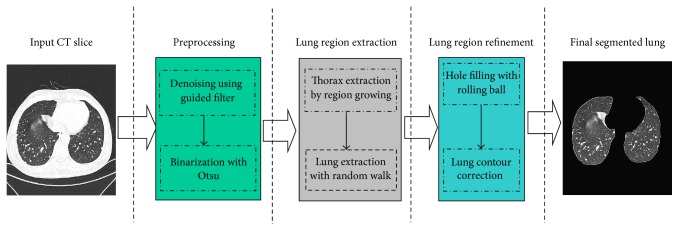
Flow chart of proposed method.

Figure 1 shows a flow of the proposed method. It effectively integrates six simple strategies in three operation steps, CT image processing, lung region initial segmentation, and lung region refinement. For the whole processing, the method began with image denoising using the guided filter [13]. Then, a threshold is selected to binarize the filtered CT images using the Otsu algorithm, and the thorax regions are then extracted by region growing. In this step, the artifacts external to the patients bodies are removed. Next, prior knowledge is used for the automated selection of foreground seeds, defined as the lung confidence region. After the estimating of seed points and that of the lung intensity range, a seed-based random walk algorithm is applied to segment lung regions from the thorax region. Finally, holes in the segmented lung region are filled with a rolling-ball algorithm, and an iterative weighted averaging and adaptive curvature threshold is used to smooth and correct the segmented lung contour on each axis slice.

Details of each operation are described in the following sections.

### 3.1. CT Image Denoising Using Guided Filter

The aim of this operation is to smooth intraregion and to preserve the interregion edges of the images, which is of benefit to the following processing, because all operations in following steps including thorax extraction by region growing and lung segmentation with random walk are sensitive to image noise. Conventional filtering methods, such as Gaussian filter, mean filter, and the median filter, often obtain poor results as they incur more edge blurring and detail loss. However, some improved anisotropic diffusion filtering methods, such as guided filters and bilateral filters, can overcome this drawback by introducing an implicit edge detection step into the filtering process to encourage intraregion smoothing and preserve the interregion edges [13]. Guided filters [13] are widely used in image smoothing as an alternative to bilateral filters, as was done in this paper. Not only can a guided filter reduce computing time compared to a bilateral filter, but also image noise which can be incorrectly regarded as lung borders in many cases can be removed from lung parenchyma. The main idea of a guided filter is to filter input images by considering the content of the guidance image. Formally, given a guidance image, a guided filter is defined as follows:

(1) $$\begin{array}{c} q_{i}=a_{k}I_{i}+b_{k},\;\forall i\in \omega_{k}, \end{array}$$

where *q*_*i*_ is a linear transformation of *I*_*i*_ in a window *ω*_*k*_ centered at the pixel (*x*, *y*) and *a*_*k*_ and *b*_*k*_ are the linear coefficients of local area *ω*_*k*_ and are supposed to be a constant. In this section, *ω*_*k*_ is assigned as 15 × 15. This local linear model ensures that *q*_*i*_ has an edge only if *I*_*i*_ has an edge. To make the difference between the output *q*_*i*_ and the input *I*_*i*_ as small as possible, the cost function *E*(*a*_*k*_, *b*_*k*_) is minimized in window *ω*_*k*_:

(2) $$\begin{array}{c} E\left(\;a_{k},b_{k}\;\right)=\overset{}{\underset{i\in \omega_{k}}{\sum }}\left(\;{\left(\;a_{k}I_{i}+b_{k}-p_{i}\;\right)}^{2}+\varepsilon a_{k}^{2}\;\right), \end{array}$$

where *ε* is a regularization parameter keeping *a*_*k*_ from being too large. In this work, the value of *ε* is assigned as 0.008 according to our experience.

Through (2), the linear coefficients *a*_*k*_ and *b*_*k*_ can be computed as follows:

(3) ak=1/ω∑i∈ωkIipi−ukp¯kσk2+ε,bk=p¯k−akuk,

where *u*_*k*_ and *σ*_*k*_^2^ are the average value and the variance of the input image *I*_*i*_ in window *ω*_*k*_. |*ω*| is the pixels number of window *ω*_*k*_. ${\bar{p}}_{k}$ is the mean of the guided image in window *ω*_*k*_. *ε* is the regularization parameter which is used to determine the intensity of changes in the pixels values.

**Figure 2:**
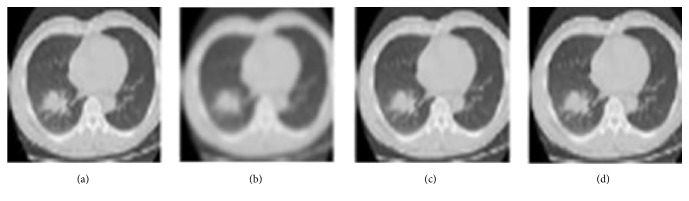
Comparison of CT image smoothing using the guided filter against other filtering methods. (a) Input CT image. (b) Smoothed CT image by Gaussian filter. (c) Smoothed CT image by mean filter. (d) Smoothed CT image by guided filter.

In our work, the guided image filter is used to filter the 3 channels of RGB image, respectively, and the guided image is selected as the corresponding original channel component.  Figure 2 shows a comparison of CT image smoothing using the guided filter against other filtering methods. It is obvious that the boundary of the smoothed image using the guided filter was clearer than when the other filters were used.

A quantitative comparison between the aforementioned filters in terms of PSNR (peak signal-to-noise ratio) was also conducted, as shown in Table 1. A higher PSNR value means that the image has high quality with less noise. It can be seen from Table 1 that the guided filter performed better than other denoise filters with the maximum PSNR of 63.1342. By contrast, the Gauss filter was inferior to both the guided filter and mean filter with a smaller PSNR of 61.4568. These results were consistent with those shown in Figure 2.

### 3.2. CT Image Binarization

In this step, Otsu's adaptive thresholding method [3] is employed to obtain a binarized CT image. The purpose of this operation is to simply follow operations for lung segmentation. For a given image, let* L* represent the grey level of the pixels [1,2,…, *L*]. By choosing a threshold at grey level* k*, the pixels are divided into object class *C*_0_ and background class *C*_1_.

Let *ω*_0_ and *ω*_1_ be the probabilities of *C*_0_ and *C*_1_ separated by a defined threshold, and let *σ*_0_^2^ and *σ*_1_^2^ be the variances of the two kinds. The variance of intrakind is defined as the weighted sum of the two variances [3], as in the following:

(4) $$\begin{array}{c} \sigma_{\text{intra}}^{2}\left(\;k\;\right)=\omega_{0}\left(\;k\;\right)\sigma_{0}^{2}\left(\;k\;\right)+\omega_{1}\left(\;k\;\right)\sigma_{1}^{2}\left(\;k\;\right). \end{array}$$

The optimal threshold* T* is calculated as the value minimizing *σ*_intra_^2^(*k*), as in the following:

(5) $$\begin{array}{c} T=\underset{k\in \left[1,L\right]}{\mathrm{argmin}}\;\;\sigma_{\text{intra}}^{2}\left(\;k\;\right). \end{array}$$

### 3.3. Thorax Extraction by Region Growing

In chest CT images, there are two main basic regions with different density distributions. The first is the low-density region, which contains background air, lungs, and airways, and the second is the high-density region, which includes the chest wall and bed and lung nodules.

**Figure 3:**
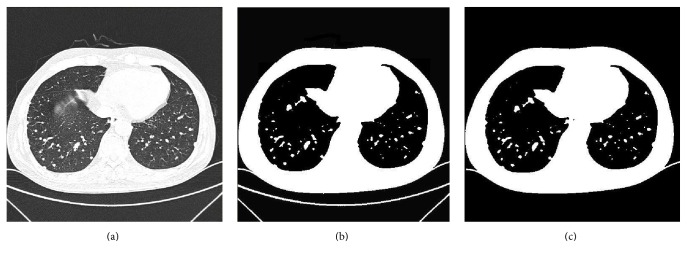
Thorax extraction by region growing. (a) Input CT image. (b) Binarized CT image. (c) Extracted thorax using region growing.

The goal of this operation is to reduce artifacts external to the patients bodies to a certain extent. Based on the density of chest CT images, to extract the thorax from the CT images in this step, region growing [4] is used in the thresholded chest CT images to discard the background. For this purpose, seed pixels are selected from the four corners of the background in each axial CT image firstly, which then grows to all pixels in the four neighborhoods. The region growing process is repeated until there are no more adjacent pixels with a lower density than threshold value* T*. Then, the background region is obtained. The background image is subtracted from the binarization CT image and then the thorax region is extracted.  Figure 3 shows an example of thorax extraction using region growing.

### 3.4. Extracting Lung from Thorax Region with Random Walk Algorithm

Considering the distribution of density of thorax tissues in CT images, random walk [12] strategy is employed for extracting lung from the thorax region in this step. Random walk is a seed-based graph method in which an image is considered a discrete object described with a weighted graph, where image pixels are taken as nodes connected by undirected edges. Taking an undirected graph

(6) $$\begin{array}{c} G=\left(\;V,E\;\right), \end{array}$$

where *V* describes the set of vertex and *E* is the set of edges, and letting *ω*_*ij*_ be the edge weight that represents the probabilities between two neighboring nodes, the weight *ω*_*ij*_ can be defined as follows:

(7) $$\begin{array}{c} \omega_{ij}=\exp\left(\;-\beta {\left(\;g_{i}-g_{j}\;\right)}^{2}\;\right), \end{array}$$

where *g*_*i*_ indexes an image feature at pixel *i* such as intensity gradients, which indicates the relationship of pixels to an image. The parameter *β* is the only parameter that can be adjusted in this method. The weights of *ω*_*ij*_ edges range from 0 to 1, letting 1 represent similar pixels and 0 represent dissimilar pixels. Given a small number of seeds in different locations, the random-walk-based lung segmentation will start its task at a pixel that reaches prelabeled seeds first by measuring the greatest transition probability. Essentially, the exact solution to the desired random walk is to minimize the Dirichlet energy with boundary conditions. The Dirichlet integral can be described as follows:

(8) $$\begin{array}{c} D\left[\;x\;\right]=\frac{1}{2}x^{T}Lx=\frac{1}{2}\overset{}{\underset{\omega_{ij}\in E}{\sum }}\omega_{ij}{\left(\;x_{i}-x_{j}\;\right)}^{2}, \end{array}$$

where the function* x* is only the critical points, which will be minima, and* L* is a Laplacian matrix described as follows:

(9) $$\begin{array}{c} D\left[\;x\;\right]=\frac{1}{2}x^{T}Lx=\frac{1}{2}\overset{}{\underset{\omega_{ij}\in E}{\sum }}\omega_{ij}{\left(\;x_{i}-x_{j}\;\right)}^{2}. \end{array}$$

Derived from the Laplacian graph expressing the image, the analysis and computation of the probabilities are obtained by resolving a set of sparse and positive definite linear equations. In random walk processing, each step usually works with previous steps independently. And then its behavior is absolute according to a transition probability matrix* L*.

**Figure 4:**
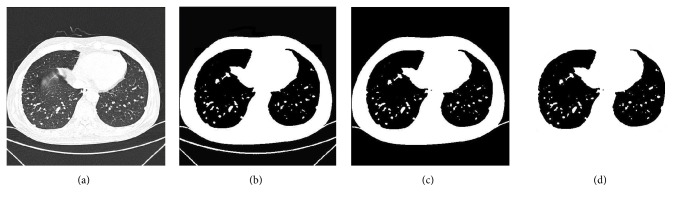
Final result for lung extraction. (a) Input CT image. (b) Binarized CT image. (c) Extracted thorax by region growing. (d) Extracted lung region by random walk.

Research shows that the random walk method demonstrates good performance in image segmentation and is sensitive to initial seeds. In CT images, the intensity of lung tissue is usually in 400 HU to 600 HU, while the chest wall, blood, and bone are usually above 100 HU [4]. Aimed at the issue mentioned above, pixels with 500 HU within the thorax region are selected as initial seeds firstly, and then a set of pixels with minimum HU values surrounding the initial seeds are sampled as seeds. Once seeds and affinity parameters for the random walk are set, lung delineation is performed. In this work, three initial seeds are automatically selected.  Figure 4 shows a final result for this step.

### 3.5. Lung Region Refinement

**Figure 5:**
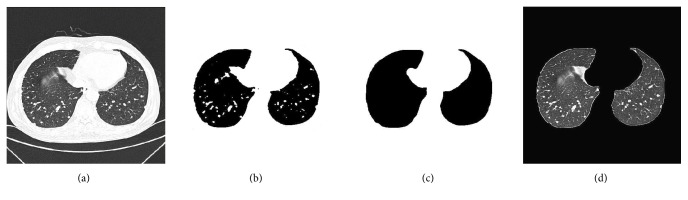
Example of final refined lung mask and final segmented lung region. (a) Input CT image. (b) Extracted lung region using random walk. (c) Refined lung segmentation mask. (d) Final segmented lung region.

As can be seen in Figure 4, it is obvious that though the lung region was extracted, there were many holes in the extracted lung regions, and parts of the regions were also excluded from the extracted lung region, which may lead to important tissue information being lost. In order to overcome such problems, holes in the segmented lung region are filled with a rolling-ball algorithm, and the contours of the segmented lungs are refined by a curvature-based correction method [14] in which a scan line search is used to calculate the curvatures of scanned points on the preliminary contour described by the random walk. In order to cut down computation time, each CT slice is scanned in the horizontal direction with a predefined interval *l* by seeking the intersection points, which is experimentally set to 3 pixels. The intersection points of the scanned edge are classified into three species, namely, the first point (*P*_*i*_^first^), the last point (*P*_*i*_^last^), and the middle point (*P*_*i*_^middle^). The middle point should be removed, as it is frequently found around the mediastinum or indentations that include the lung nodules. Thus, only the first and last intersection points are retained as they correspond to the lateral and medial lung contours. The curvature of the first and the last intersection points is computed as follows:

(10) ki=xi−1−xi×yi−1−2yi+yi+1×yi−1−yi×xi−1−2xi+xi+1xi−1−xi2+yi−1−yi23/2,

where (*x*_*i*_, *y*_*i*_) denotes an intersection point on *i*_th_ search line. Similarly, (*x*_*i*−1_, *y*_*i*−1_) and (*x*_*i*+1_, *y*_*i*+1_) are the same on the previous and following scan lines. For most natural images, taking into account high curvatures located at small perturbations, such as at the base and areas of normal lungs, the differences are used to rule out unnecessary points.  Figure 5 shows an example of the final refined lung mask and final segmented lung region. As seen, the proposed method worked well.

## 4. Experiments

In this section, the clinical materials used in this work and the evaluation criteria are described. Then, the detailed results are presented, which include the visualization of segmentation errors and quantitative and statistical accuracy comparisons. Finally, the issues and limitations that were observed in the experiments are discussed. All methods were implemented in Matlab and tested using a 2.3 GHz Intel Core i3 computer with 2 GB RAM.

### 4.1. Materials

The database used in this study consisted of 23 CT scans, including a total of 883 2D slices, which were acquired using MDCT scanner (GE Light-Speed Ultra, Milwaukee, WI, USA) with 120 kVp and 100 mA in the medical school of Xian Jiaotong University. The number of slices per scan is about 38 slices per scan. Each CT slice had an image matrix of 512 by 512 (16-bit depth) pixels. Pixel size ranged between 0.625 mm and 0.742 mm, with a mean value of 0.692 mm, depending on the physical size of the patient.

### 4.2. Evaluation Method

Quantitative evaluation of lung segmentation is important because it not only provides a reliable basis for clinical application but also indicates its relative performance with respect to other used methods [4]. However, conducting an evaluation of a lung segmentation method is still difficult. One reason is that the true lung boundary is unknown, and the reference standard often refers to several experts consensus [6]. In this study, the reference standard was produced in the following way to address this issue: all lung contours were first manually marked by an experienced radiograph expert and then reviewed by another radiologist. If the opinion of the second radiologist was different from that of the first one, the lung contours were corrected by the two radiologists under collaboration and the results were used as the reference standard. Although only limited radiologists involved in the manual segmentation might lead to bias, the difference between the lung boundaries obtained by this method and the reference standard can reflect the errors of the proposed method with respect to an expert.

Another reason for difficulty is that even though there are many metrics used to evaluate a lung segmentation method, such as dice similarity coefficient, jacquard similarity, false positive rate, and false negative rate, they do not provide both local and global impressions of the segmentation performance [6]. Because of this, the following three metrics for measuring the segmentation performance of the proposed method were employed: (1) oversegmentation rate, (2) undersegmentation rate, and (3) the average of absolute border distance. The experiments showed that these three metrics not only demonstrated an overview of the oversegmentation and undersegmentation but also confirmed the whole statistical distribution of segmentation error distances.

The oversegmentation rate is termed as the number of voxels in a segmented region which are included as part of the ROI but are not in the reference standard. Let *V*_auto_ represent the volume of the binary mask generated using the proposed approach and let *V*_mannual_ be the volume of the reference standard. The oversegmentation rate of OR(*V*_auto_, *V*_mannual_) can be found using the following:

(11) $$\begin{array}{c} \text{OR}\left(\;V_{\text{auto}},V_{\text{mannual}}\;\right)=\left|\;\frac{V_{\text{auto}}/V_{\text{mannual}}}{V_{\text{mannual}}}\;\right|, \end{array}$$

where *V*_auto_/*V*_mannual_ represents the relative complement of *V*_auto_ in *V*_mannual_. Similarly, the undersegmentation rate of UR(*V*_auto_, *V*_mannual_) is defined as the relative lung volume amount which is regarded as lung tissue in the reference standard but not in a segmented image region with an automatic segmentation method:

(12) $$\begin{array}{c} \text{UR}\left(\;V_{\text{auto}},V_{\text{mannual}}\;\right)=\left|\;\frac{V_{\text{auto}}/V_{\text{mannual}}}{V_{\text{mannual}}}\;\right|. \end{array}$$

The average of the absolute border distance (ABD) is a statistical measurement of the fitting between the lung surfaces generated by a segmentation method and the lung surfaces in the reference standard. It is used to measure the spatial similarity between the lung boundaries generated by a segmentation approach and that of the reference standard. The shortest distance between a point on the lung surface obtained by the proposed algorithm and the lung surface of the reference standard was used to generate the absolute border distance.

### 4.3. Qualitative Results

**Figure 6:**
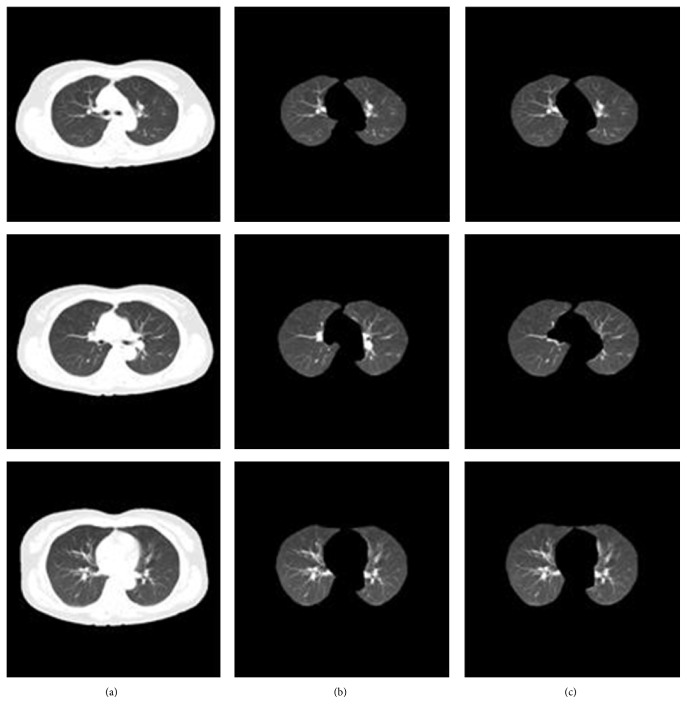
Comparisons between ground truth and the proposed method. (a) Input CT images. (b) Ground truth. (c) Results using proposed method.

Figure 6 shows the results between ground truth and the proposed method.  Figure 6(a) shows the input CT slices. The ground truth is in Figure 6(b), which was manually marked by an experienced radiograph expert.  Figure 6(c) displays the segmented results using the proposed method. As can be seen, the proposed method's segment results were closest to the ground truth, which indexes the effectiveness of the proposed method.

**Figure 7:**
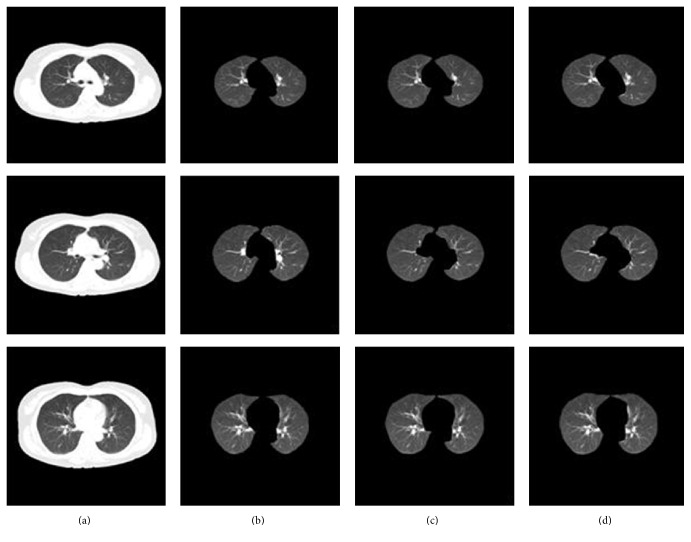
Comparison of segmentation results. (a) Original CT images. (b) Active-contour-based method. (c) Region growing-based method. (d) The proposed method.

A comparison of the proposed approach with two often-used state-of-the-art lung segmentation methods, the active-contour-based method [9] and the region growing-based method [4], is shown in Figure 7.  Figure 7(a) shows the input CT images, and Figure 7(b) shows the segmented results using the active-contour-based method. In Figure 7(c), the region growing-based method is shown. The proposed method's segmentation results are shown in Figure 7(d). From Figure 7(c), it can be seen that even though the lung boundaries are well smoothed, part of the pleural regions in the mediastinum was excluded. In Figure 7(b), although no lung regions were excluded, parts of nonlung regions were erroneously included. However, Figure 7(d) shows sufficient pleural nodule regions and diffuse areas are also included. This indicates that the proposed method exhibits a more powerful discriminating ability compared to other methods.

### 4.4. Quantitative Results

Table 2 shows comparisons between the proposed method and two state-of-the-art techniques, the region growing method and the active contour method, to the manually defined ground truth using prior-mentioned metrics. It can be seen that the average of the absolute border distance, the oversegmentation rate, and the undersegmentation rate of the proposed method were 0.62 mm, 1.87%, and 2.36%, respectively. These results were better than those of the region growing-based method (0.72 mm, 2.1%, and 2.7%) and the active-contour-based method (0.64 mm, 1.9%, and 2.38%). This indicated that the proposed method achieved more accurate and robust results than the other approaches.

## 5. Discussion

**Figure 8:**
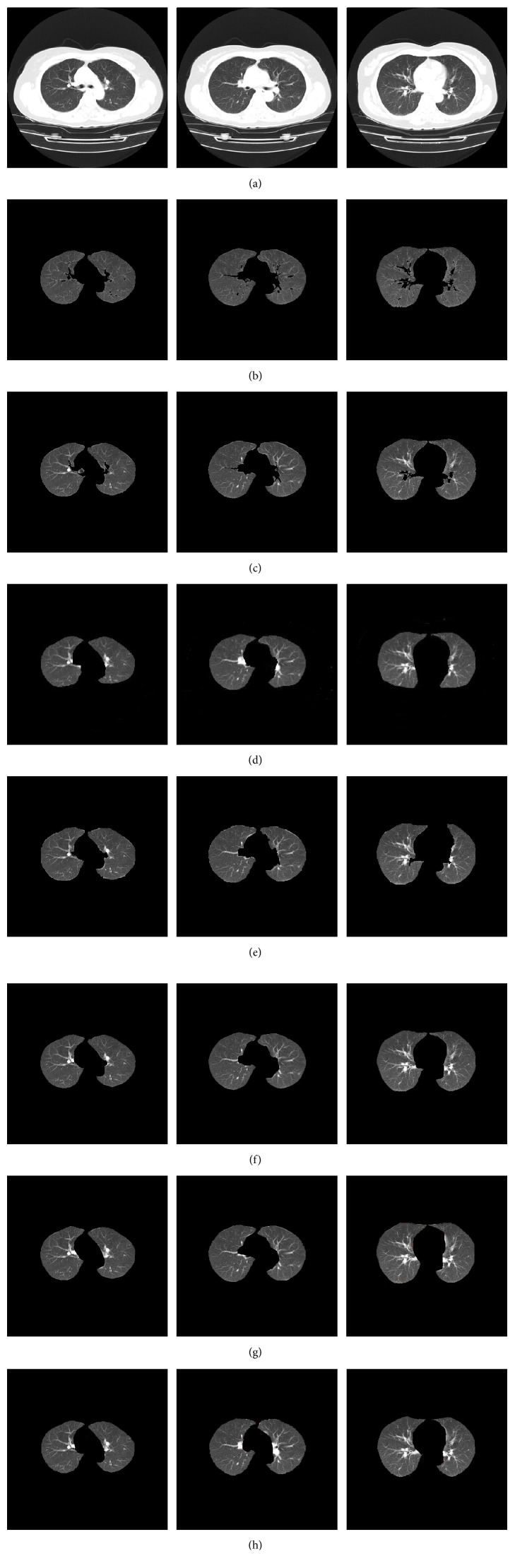
Comparisons of segmentation results. (a) CT images. (b) Threshold method. (c) Region growing method. (d) Active contour method. (e) Random walk method. (f) Active contour with curvature-based correction method. (g) Proposed method. (h) Reference standard.

As an integration of multiple simple image segmentation strategies, the method proposed in this paper possesses several advantages over single segmentation strategy methods, as illustrated in Figure 8.  Figure 8(a) shows input CT slices, Figure 8(b) shows the results obtained with the threshold-based method, Figure 8(c) is the region growing-based method, Figure 8(d) is the active-contour-based method, Figure 8(e) is the random walk method, Figure 8(f) is the active contour and curvature correction method, and Figure 8(g) shows the segmented results using the proposed method.  Figure 8(h) shows the reference standard. As can be seen in the images, compared with the reference standard, the main trachea was excluded from the segmented lung area using the threshold-based method. The region growing-based method made the lung edge rough, and good segmentation results of lung regions were not achieved due to the limiting of growth rule. In the evolution of the active contour curves, the diffuse area was left out. The random-walk-based method produced inaccurate segmentation results. As can be seen from the results of the active contour and curvature-based correction method, the lung boundary was smoothed with undersegmentation in the mediastinum. By comparison, the developed approach demonstrated a more powerful discriminating ability and included sufficient pleural nodule regions and diffuse areas.

A quantitative comparison between the segmentation results obtained with an assemblage of multiple segmentation strategies against other segmentation strategies was also performed using the overlap ratio between the manually outlined contours and computer-defined outlines, as shown in Table 3. The overlap ratio can be defined using the following:

(13) $$\begin{array}{c} \text{overlap ratio}=\frac{N_{\text{TP}}+N_{\text{TN}}}{N_{\text{TP}}+N_{\text{TN}}+N_{\text{FP}}+N_{\text{FN}}}, \end{array}$$

where *N*_TP_ represents the number of correctly segmented pixels in a lung region, *N*_TN_ stands for the number of correctly segmented pixels in the background area, and *N*_FP_ and *N*_FN_ are the missegmented lung regions and background area, respectively. A high overlap ratio indicates accurate segmentation results. As can be seen from Table 3, the average overlap ratio obtained using the proposed method was 98.4%, whereas that obtained using the threshold-based method was 94.1%, the region growing overlap ratio was 95.3%, the active contour was 94.4%, the active contour with a curvature-based correction method was 95.8%, and the random-walk-based method was only 93.8%. These results were consistent with those shown in Figure 8. The conclusion from these results is also consistent with the outcome mentioned in Table 2.

**Figure 9:**
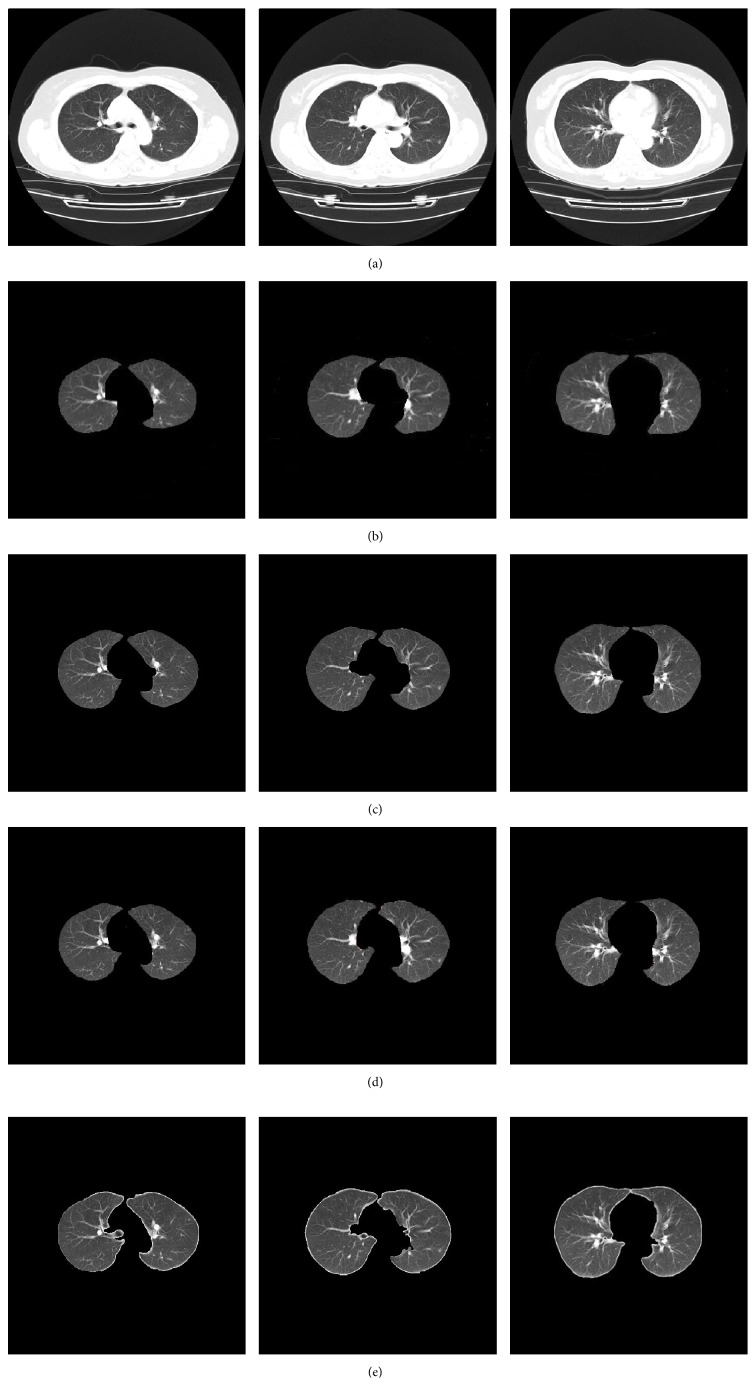
Comparisons of segmentation results. (a) Original CT images. (b) The method proposed in [11]. (c) The graph-cut based method presented in [5]. (d) Our method. (e) Reference standard.

A comparison between our method and some recently published, independent methods, such as the graph-cut based method presented in [5] and the method proposed in [11], is shown in Figure 9 and Table 4. It can be seen from Figure 9 that the segmented lung images are very similar, and the difference between the segmented lung image with our method and that with the graph-cut based method presented in [5] and also that with the method proposed in [11] are too tiny to observe.

Table 4 shows the comparison in terms of the oversegmentation rate and the undersegmentation rate. As can be seen, the oversegmentation rates of our proposed method, of the graph-cut method [5], and of the method proposed in [11] are 1.87%, 1.88%, and 1.86%, respectively, whereas the undersegmentation rates of these are 2.36%, 2.34%, and 2.37%, respectively. This indicated that the segmentation accuracies of the three methods mentioned above are similar. This conclusion is consistent with the outcome demonstrated in Figure 9.

A comparison of running time for all methods mentioned above is given in Table 5. It can be seen that the proposed method requires longer working times (1.68 s for one slice segmentation) compared to previously well-established methods, such as threshold-based method (0.28 s for one slice segmentation), region growing (0.38 s for one slice segmentation), active contour (0.54 s for one slice segmentation), random walk (0.58 s for one slice segmentation), and active contour with curvature correction (0.64 s for one slice segmentation), whereas compared to the graph-cut based method [5] (3 s for one slice segmentation) and the method proposed in [11] (2.58 s for one slice segmentation), the running time of our method is significantly short. What is the reason for this? For the proposed method and previously well-established methods, the reason is that the new method is an integration of six simpler image segmentation strategies meaning more time is needed to carry out all the steps. For the proposed method and the graph-cut based method, the reason is that the use of expectation maximization to calculate the weight that each pixel belongs to the foreground object leads to a longer running time than that of our proposed method. For the proposed method and the method proposed in [11], the reason is that the fuzzy c-means method which is used for lung identification might lead to a longer running time.

Considering the compromise between the accuracy of the segmented results and the computing time of the whole method, it is obvious that our proposed method is more efficient.

## 6. Conclusions

In this paper, an assemblage of several simple image segmentation strategies was proposed for segmenting lung regions in chest CT images. The effectiveness of this approach was demonstrated on 23 CT scans, and the results were compared to the manual segmentations of an expert and results obtained with the two most esteemed techniques. Experimental results showed that this method was more accurate in lung segmentation compared to other methods.

It should be noted that accurate segmentation of lung regions in the presence of severe pathologies, such as lung cancer, is still a challenging task. Future work will mainly focus on the segmentation algorithm of lung tissue characterized with severe abnormalities.

## Figures and Tables

**Table 1:** Quantitative comparison of different denoise filters.

	Ground-truth marker	The number of locations recognized by the system
Images during day	100	98
Images during night	100	90

**Table 2:** Quantitative comparison of segmentation results.

Methods	OR	UR	ABD (mm)
Region growing	2.1%	2.7%	0.72
Active contour	1.9%	2.38%	0.64
Proposed method	1.87%	2.36%	0.62

**Table 3:** Quantitative comparison between segmentation results.

Methods	Overlap ratio (%)
Threshold	94.1
Region growing	95.3
Active contour	94.4
Random walk	93.8
Active contour with curvature correction	95.8
Proposed method	98.4

**Table 4:** Quantitative comparison of segmentation results in terms of the oversegmentation rate and the undersegmentation rate.

Methods	OR	UR
Method in [11]	1.86%	2.37%
Method in [5]	1.88%	2.34%
Proposed method	1.87%	2.36%

**Table 5:** Working times of different methods.

Methods	Average running time (seconds)
Threshold	0.28
Region growing	0.38
Active contour	0.54
Random walk	0.58
Active contour with curvature correction	0.64
Method in [11]	2.58
Method in [5]	3
Proposed method	1.68
